# Anaphylactic reaction 5 minutes after the start of surgery: a case report

**DOI:** 10.1186/s13104-015-1060-9

**Published:** 2015-04-01

**Authors:** Manuela Malsy, Richard Leberle, Katharina Ehehalt, Barbara Sinner, Jonny Hobbhahn

**Affiliations:** Department of Anesthesiology, University Medical Center Regensburg, Franz Josef Strauss Allee 11, 93053 Regensburg, Germany

**Keywords:** Natural latex rubber, Anaphylaxis, Anaphylactic shock, Children, Perioperative complications, Anesthesia

## Abstract

**Background:**

Natural latex rubber products have been known to cause severe anaphylactic reactions during surgery. Even 25 years after the first description of anaphylactic reactions in the literature, natural latex rubber products are still used in pediatric surgery.

**Case presentation:**

The following article describes the case of a healthy 4.5-year old Caucasian boy who simultaneously developed severe hypotension, tachycardia and bronchospasm during surgery for congenital strabismus sursoadductorius under uneventful anesthesia. An allergy test conducted afterwards showed natural latex rubber as the trigger for this severe intraoperative anaphylactic reaction.

This case was special because of the absence of any previous clinical or anamnestical evidence of natural latex rubber allergy. The fact that the child had been previously exposed to natural latex rubber – because the boy’s mother used disposable gloves for her work as a cosmetician at home – was only discovered later. Such contact may have had a slight sensitizing effect that manifested after the initial contact with the conjunctiva through the surgeon’s natural latex rubber gloves.

**Conclusion:**

Natural latex rubber products have caused severe anaphylactic reactions time and again. Diagnosis is impeded by the highly variable clinical symptoms of anaphylaxis, the non-responsivity of patients, anesthesia-induced changes in blood pressure, surgical drapes, and blood loss. Therefore, use of alternative products and implementation of the right course of action in clinical routine seems to be even more important than raising awareness for allergies to natural latex rubber.

## Background

The wearing of natural latex rubber gloves in healthcare protects both patients and health carers from infections and contaminations. Apart from high wearing comfort, natural latex rubber gloves are generally characterized by unequaled physical qualities, such as [1]. However, natural latex rubber also has the disadvantage of causing severe anaphylactic reactions. The first case of allergic reaction was documented by Grete Stern in 1927 who described a patient with symptoms of urticaria and intermittent Quincke’s edema that lasted six months. The patient, who had been wearing dentures with a natural latex rubber plate, showed swelling of one side of her face, both her eyelids or lips, and life-threatening glottal edema. Once the dentures were removed, the Quincke’s edema abated, and the patient was subsequently symptom-free. With repeated exposure, the described symptoms reoccurred. After replacement of the natural latex rubber plate of the dentures with a metal plate, the patient remained symptom-free. Thus, an allergic reaction to natural latex rubber was assumed [2].

The highly increased use of disposable gloves in healthcare is held responsible for the rising prevalence in allergic reactions to natural latex rubber. This increased use was triggered by the appearance of the first HIV (human immunodeficiency virus)-infections and the desire to be protected against blood transmitted diseases. In the year 2000, United States authorities estimated that 8% to 12% of healthcare professionals were sensitized to natural latex rubber products in contrast to only 1% of the general population [3]. The first cases of children showing an intraoperative anaphylactic reaction to natural latex rubber were reported in 1989 [4].

Up to now, natural latex rubber is still the second most common trigger for anaphylactic reactions during anesthesia [5]. An even higher number of allergic reactions, particularly in adults, are caused by muscle relaxants [6]. Clinical symptoms as well as their manifestation vary and depend on the type and extent of exposure to an allergen and a person’s individual sensitivity. Natural latex rubber causes two different kinds of allergies; the delayed type IV reaction, in which the allergen-specific T-cells are activated, and the immediate type I reaction [7]. After previous sensitization, a second contact may result in the activation of mast cells and the release of histamine, leukotriene, and prostaglandin by endogenous immunoglobulin E (IgE)-antibodies only minutes or even just seconds after the detection of antigens [8]. Allergic symptoms include local reactions, such as contact urticaria, and mucosal symptoms, such as rhinitis, conjunctivitis, and bronchial asthma, but allergic shocks with cardiovascular reactions and bronchospasms are rare [9]. A considerable number of patients do not even show the common symptoms, such as urticaria and erythema [10]. Skin or mucosal contact to natural latex rubber proteins is the trigger for the appearance of symptoms [11].

## Case presentation

The patient was scheduled for surgery because of congenital strabismus sursoadductorius of the left eye. At the day of surgery, the 4.5-year old child was normally developed. The boy was Caucasian, weighed 19 kilograms, and had no known history of pre-existing medical conditions or allergies (ASA I). Premedication was adequately administered with midazolam juice per os and EMLA dressings on the back of both hands. Intravenous anesthesia with remifentanil and propofol was started without any complications. Airways were secured with a laryngeal mask size 2. Anesthesia was maintained with remifentanil and sevoflurane. The patient received adequate doses of dexamethasone and ondansetrone to prevent postoperative nausea and vomiting as well as piritramid and ibuprofen suppositories for post-surgical pain management.

Surgery was started 25 minutes after the initiation of anesthesia. No complications or reactions to anesthesia occurred during that period. After another 5 minutes, peripheral oxygen saturation and tidal volume dropped suddenly, and severe hypotension and tachycardia occurred. Because of suspected anaphylaxis with massive bronchospasms, guideline-oriented treatment was started immediately consisting of histamine H_1_- and H_2_-blocking agents (dimetinden [0.1 mg/kgKG], ranitidin [1.0 mg/kgKG]) and prednisolone (4.0 mg/kgKG), fractional adrenaline or noradrenaline, and volume replacement (20.0 ml/kgKG). Swelling of the epiglottis was detected during the switch to an endotracheal tube (ID 4.5) to secure adequate respiration. Arterial cannulation for continuous invasive blood pressure measurement and blood gas analysis was conducted, and inhalation of salbutamol was started.

45 minutes after the initiation of the above-mentioned measures, the patient showed cardiorespiratory stabilization. Therefore, surgery − that had been scheduled for 90 minutes − was continued without any further incidents. A 7 mm revertive positioning of Musculus rectus internus, a 5 mm revertive positioning of Musculus obliquus inferior, as well as a 5 mm folding of Musculus rectus externus of the left eye was administered. After surgery, the patient was extubated without any problems. Because cardiorespiratory conditions were stable and glottal swelling was significantly reduced, the patient was transferred to the recovery room. The patient woke up only a few hours later, breathing spontaneously, and was transferred to the pediatric intensive care unit in good cardiovascular condition. IgE levels from blood drawn after surgery were distinctly increased at 835 IU/ml (norm < 60 IU/ml) and validated the suspected diagnosis of an allergic reaction. Indication marker for natural latex rubber (k82) showed an increased level of 11.30 kU/l that corresponded to CAP-class 3, thus confirming an allergic reaction to natural latex rubber.

## Discussion

Severe allergic reactions to natural latex rubber have been documented in the literature since 1990. Since then, the incidence of allergic reactions could be considerably decreased by avoiding intraoperative rubber exposure, which is still the most important measure to reduce such reactions in children. Despite this knowledge, natural latex rubber products are still in use because of the higher costs of latex-free products, such as nitrile and vinyl, and their inferior quality. Incomplete manufacturers’ specifications pose yet another problem in identifying products containing latex.

Examples for products containing latex for medical use:

- gloves
- medical bottle tops
- catheters
- blood pressure cuffs
- ventilation bags
- tourniquets
- adhesive tapes

Further reasons for reducing the use of natural latex rubber were high-risk groups, such as patients with atopic syndrome or Spina bifida, surgery in infancy, and medical personnel [12]. However, prevalence is still high, and allergic reactions to natural latex rubber represent potentially life-threatening intraoperative complications [13]. Mortality rates for allergic reactions to natural latex rubber during anesthesia still amount to 5% to 7% [14]. Patients with atopic syndrome show a significant predisposition towards sensitization to natural latex rubber, meaning a constant repeated contact with materials containing rubber [1]. Many affected children have had multiple surgeries because of Spina bifida or anomalies of the urogenital region in early infancy and thus high exposure of natural latex rubber [15].

The medical history of the patient of this case report was inconclusive. The fact that the child had been previously exposed to natural latex rubber – because the boy’s mother used disposable gloves for her work as a cosmetician at home – was only discovered later. Early exposure to natural latex rubber is a relevant factor for developing an allergy to natural latex rubber in later life [6]. Such contact may have had a slight sensitizing effect that manifested after the initial contact with the conjunctiva through the surgeon’s natural latex rubber gloves. Until that moment, even though unwittingly, every product used was free of natural latex rubber. Despite the administration of an adequate dose of dexamethasone − that presumably prevented an even worse reaction − a significant allergic reaction could be observed.

In case of an intraoperative anaphylactic reaction, it is critical to first stop the contact with the allergen. The following list shows a short overview of necessary acute measures:

Acute measures:

- Stop contact with the allergen:
  - All personnel wearing latex gloves have to leave the operating theater immediately or as quickly as possible.
  - Gloves have to be taken off outside, and theater clothes must be changed.
  - The removal of all items containing latex is imperative!
- Personal recruitment of personnel, such as physicians and nurses.

Additional acute measures for stabilizing a patient are to be initiated by means of the ABCDE scheme. Priorities are securing airways, sufficient oxygen levels, and stable circulation with the help of increased volume intake and fractional adrenaline. Corticosteroids and antihistamines need to be administered simultaneously.

Acute measures based on the ABCDE scheme:

A. Airways: In case of swelling, administer nebulized adrenaline via a mask, intravenous corticoids, and intubate if necessary. Beware complications due to swelling.
B. Breathing: Respiration with sufficient FiO_2_, bronchoconstriction needs to be treated with nebulized bronchodialators.
C. Circulation: Additional venous catheters, volume substitution, adrenaline boluses, and cardiopulmonary resuscitation if necessary.
D. Disability: Second-line-therapy with H_1_/H_2_- antihistamine.
E. Exposure: Further physical examinations – reevaluation.

For preventing allergies to natural latex rubber, premedication with corticoids and antihistamines has been suggested. *Caffarelli et al.* advised against relying on premedication because of the lack of documented evidence on its effectiveness in preventing allergic reactions to natural latex rubber [16]. The cost of extensive pre-surgery screening is also not justified for every patient [17]. Preoperative testing by means of the ‘skin prick method’ is not required but can aid diagnosing, particularly for patients with Spina bifida (up to 44% show latex allergies), dysplasia of the genitourinary tract, and atopic dermatitis, as well as for patients with occupational exposure to latex and allergies to Ficus elastica or food (kiwis, figs, papayas, and chestnuts). The authors of this case report recommend routine allergy assessment by means of a standardized questionnaire to preoperatively detect high-risk patients.

Questionnaire:

- Do you or your child have any known allergies?
- Have you or your child ever shown allergic reactions to food, such as kiwis, figs, papayas, or chestnuts?
- Have you or your child been exposed to latex products, such as balloons, protective gloves, condoms or sealants?
- Have you or your child ever had surgery before?

A standardized procedure is recommended if the answers of the questionnaire result in a suspected high allergy risk to latex products.

In case of a suspected risk or a minor allergic reaction:

- Immediate information of all theater personnel.
- Patient must be the first undergoing treatment on the day of surgery, and ‘latex allergy’ must be documented on the theater schedule.
- All natural latex rubber products must be removed from the theater.

In case of presumably extreme reactions:

- Day before surgery:
  - Measures as described above, additionally:
  - Thorough cleaning of theater and equipment.
  - Theater must not be used during the night.
  - Doors must be kept closed.
- Day of surgery:
  - Theater personnel must not have any contact with natural latex rubber products.
  - The theater may only be entered with clean clothes.
  - No administration of medications releasing histamines.
  - Provision of prophylactic medication: H_1_- and H_2_-blocking agents, glucocorticoids.
  - Emergency medication must be prepared and ready for use.

## Conclusions

Avoiding natural latex rubber products is still the most important measure to reduce allergic reactions in children. A task committee could be formed to compile a list of materials containing latex in the theater and to issue a standardized questionnaire on preoperative assessment and a structured written course of action. Establishing standardized operating protocols in the surgical process guarantees a structured reaction in case of perioperative anaphylaxis [10]. Such processes should be written down in a standard operating procedure (SOP) and repeatedly exercised in emergency training.

## Consent

Written informed consent was obtained from the patient's parents for publication of this Case Report and any accompanying images. A copy of the written consent is available for review by the Editor-in-Chief of this journal.

## Abbreviations

HIV: Human immunodeficiency virus
IgE: Immunoglobulin E
ASA: American society of anesthesiologists-physical status
EMLA: Eutectic mixture of local anesthetics
ID: Inside diameter
kgKG: Kilograms per bodyweight
CAP: Capacity classes
IU: International unit
FiO_2_: Inspired oxygen concentration
SOP: Standard operating procedure
